# Hygiene and biosecurity conditions of initial examination on-spot in Portugal: One step toward game meat safety

**DOI:** 10.14202/vetworld.2023.882-887

**Published:** 2023-04-29

**Authors:** Ana Carolina Abrantes, Maria Pureza Ferreira, Zita Ruano, Bruno Vinhas, Yolanda Vaz, Madalena Vieira-Pinto

**Affiliations:** 1 CECAV-Animal and Veterinary Research Centre, UTAD, Vila Real, Portugal; 2 CIISA, Faculty of Veterinary Medicine, Lisbon University, Lisbon, Portugal; 3 Department of Veterinary Sciences, Trás-os-Montes e Alto Douro University, Vila Real, Portugal; 4 Associate Laboratory for Animal and Veterinary Sciences (AL4AnimalS), Portugal

**Keywords:** food safety, good practices, public health

## Abstract

**Background and Aim:**

Due to the particularities of the first steps of the game food chain, large game species are shot, bled, and handled in collection points (spot of evisceration and initial examination in the field). These steps of the game meat chain affect the microbiological quality of this type of meat, thus posing a risk to consumers. This study aimed to characterize the collection points in terms of central hygiene and biosecurity procedures/requirements.

**Materials and Methods:**

One survey with 16 questions was applied in 95 hunting areas throughout Portugal. It was a convenience sample obtained by direct visualization on-spot procedures. Four categories were characterized in the survey: Initial examination (performance assiduity and type of operator performing it), hygiene requirements on-spot (floor, ceiling, water, and electricity), biosecurity procedures such on initial examination (use of personal protective equipment as gloves, glasses, mask, and specific clothes), and by-products disposal (destination and packaging of by-products).

**Results:**

Sixty percentage (n = 57) eviscerated the carcasses and performed the initial examination on-spot. Moreover, most of the time (n = 71), the initial examination was carried out by veterinarians. However, the category that showed the best results was those related to the biosecurity procedures on initial examination, mainly with the use of the individual protective material (e.g., regular use of disposal clothes and specific clothes). Concerning the questions about the disposal of by-products, 66 game managers say that this was done correctly (69%), being the majority destination of the inspected carcasses was the burial (64%; n = 47).

**Conclusion:**

This survey demonstrates an immediate need in all this problematic of the standardization of hygiene and biosecurity requirements of the collection points, which requires uniform application of rules. There is a lot of resistance and limitations to the inclusion of these requirements in collection points, due to lack of structural and financial conditions. However, training all those involved in the hunting area (hunters, game managers, authorities, etc.) creating rules that promote hunting food security and setting limits on the microbiological criteria of game meat are hot points to consider in the future.

## Introduction

Large game hunting is considered a primary production and has a relevant economic impact in some countries, such as Italy [1], Spain, Portugal [2], and Austria [3]. Due to the fact that, game meat is a product with excellent attributes: tasty, high nutritional value, and low-fat content [4]. However, there are potential risks associated with its consumption; some favored by the mode of slaughter, evisceration conditions, carcass storage, and ante- and postmortem inspection, which can compromise the microbiological requirements of game meat [1, 5]. Thus, the safety of game meat depends on effective on-spot sanitary control [6, 7].

Due to the particularities of the first steps of the game food chain, large game species are shot, bled, and eviscerated in the field. These procedures that occur initially in the game meat chain affect the microbiological quality of the meat and are recognized as the first level of contamination of the final carcasses [1]. However, microbiological contamination comes from the animal itself, the external environment, and the operators [8]. Apart from the phases of pre-collection of the carcasses from the shotting field, the hygiene and microbiological contamination of the hunted carcasses depends on the requirements of the game collection points (the intermediate spot where it is eviscerated and undergoes the initial examination as soon as possible and transported to a game handling establishment) and the good hygiene practices during the evisceration and the initial examination, due the fact that there is no access to hygienic conditions like those present in modern slaughterhouse facilities [9].

In Europe, the initial examination must be systematic and carried out by an operator legally trained, according to Reg. 853/2004, or a trained person or a veterinarian [6]. Proper training for these operators should be given for them to have sufficient knowledge about hunting diseases to identify the pathological lesions at the scene of an initial examination and to ensure correct hygiene procedures and effective elimination of unfit meat and offal [3, 10]. In countries like Portugal, specific rules exist [11]. The mandatory initial examination of a large game by a veterinarian exists in a particular area delimited along the border with Spain, coinciding with the area at risk of Tuberculosis (Notice No. 1 of tuberculosis in the large game). Thus, although not mandatory in the rest of the country, it is advisable to conduct an initial examination in any hunting act, which can be carried out by a trained person. During this process, biosecurity measures and hygiene requirements are crucial points in terms of food safety and zoonoses prevention in the case of the large game meat chain. To keep operators’ health safe, some requirements must be met, such as using protective material like disposal clothes, gloves, disposable protection of shoes, glasses, and masks. In the case of hygiene and biosecurity requirements of the initial examination spot [2], to avoid cross-contamination between carcasses and ensure environmental health, the spot must be equipped with electricity, potable water, means of waterproofing the soil, and means of disinfection of knives and equipment [11, 12]. A safe way of disposal/disposal of by-products and unfit meat must also be provided by the organizers of the hunting act [3].

Microbial conditions of the game meat depend on many factors: environmental, carcass handling, and processing. In this measure, operators, primarily hunters and game managers, are responsible for hygiene in the different phases, killing, evisceration, initial examination, and further handling and trading of the carcasses from the collection to the chilling point [3, 10, 13]. This point is also of great importance in terms of food safety and the operator’s health safety of the operator once they know how to mitigate the risk of acquiring zoonotic agents.

This study aimed to characterize the collection points (spot of evisceration and initial examination) in terms of main hygiene and biosecurity procedures/requirements.

## Materials and Methods

### Ethical approval and Informed consent

This survey does not include questions to professionals (game managers or veterinarians), or hunters. It only includes direct observation of the biosecurity and hygiene conditions and requirements of the collection points. The responsible organization of the driven hunts and respective collection points were informed about the study, and all of them signed an informed consent form authorizing the direct observation of the conditions analyzed in the study.

### Study period and location

The study was conducted during three hunting seasons 2020-2021, 2021-2022 and 2022-2023, specifically between the months of October and February. The location of the studied 95 hunting areas is dispersed throughout Portugal: 21 in the North, 38 in the Center and 36 in the South.

### Sampling

Ninety-five Portuguese hunting areas were visited: During biosecurity assessment visits (n = 55) or at the end of driven hunts in the hunting season (n = 40). It was a convenience sample obtained by directly visualizing the spot and procedures.

Of these 95, 40 are in the risk area of tuberculosis for the large game (Notice Nº1), where it is mandatory to carry out an initial examination on-spot after the driven hunts and other specific rules, such as:

- “…There must be a place to the evisceration of hunted animals… with proper conditions to carry out that task…”;
- There must be presence of a veterinarian responsible for the initial examination of every harvested animal presented in the collection points to come up with one of the following results:
  1. Animals which present alterations that may suggest a health risk must go to by-products or if the hunting area requests to a specific establishment for game meat preparation to a final decision been taken;
  2. Animals that do not pre-sent present alterations that may suggest a health risk go to self-consumption or to a specific establishment for game meat preparation to be inspected and placed in the market.

### Evaluation of hygiene and biosecurity conditions: On-spot survey

In each driven hunt, the large game hunted were collected and gathered at the collection point. Sometimes, the sanitary assessment of game meat is carried out through the initial examination that a veterinarian or a trained person can carry out. The initial examination procedure was based on Regulation (EC) 854/2004, which sets rules for official controls of animal-origin products intended for human consumption and consists of questions to the hunters about abnormal behavior, external carcass examination, and internal carcass examination.

In this study, a systematic evaluation of game meat preparation conditions in collection points was performed. Collection points’ information about evisceration, initial examination, and by-products disposal was collected through an on-spot survey. The generic on-spot study consists of 16 questions described in Table-1 (e.g., question: possible answers) divided into four categories (initial examination, hygiene requirements on-spot, biosecurity procedures on initial examination, and by-products disposal).

**Table-1:** On-spot survey with 16 questions (Four categories).

Category 1: Initial examination (Four questions)	
1. Percentage of the total of animals hunted with official inspection	>50%/<50%/0%
2. Are hunted animals eviscerated on the territory of the hunting area?	never/always/sometimes/not applicable
3. Are animals hunted in this hunting area subject to initial examination on-spot?	Never/always/sometimes/not applicable
4. If it is performed, who does the initial examination?	Trained veterinarian/veterinarian without specific formation/trained person/hunter without specific formation/not applicable
Category 2: Hygiene requirements on-spot (5 questions)	
1. Location of the collection point (where the carcasses are eviscerated and/or the initial examination is conducted)	Inside the hunting area/outside the hunting area/not exist a specific spot
2. Is the spot paved?	No/yes/not applicable
3. Is the spot covered?	No/yes/not applicable
4. Does the spot have potable water?	No/yes/not applicable
5. Does the spot have electricity/light?	No/yes/not applicable
Category 3: Biosecurity procedures on initial examination (Four questions)	
1. Use of disposable gloves	No/yes/not applicable
2. Use of mask	No/yes/not applicable
3. Use of protective glasses	No/yes/not applicable
4. Use of specific clothes or disposable suits	No/yes/sometimes/not applicable
Category 4: By-products disposal (3 questions)	
1. Is the collection point cleaned and disinfected?	No/only clean/clean and disinfected/not applicable
2. Carrying out proper disposal of by-products of hunted animals after evisceration and initial examination?	No/yes/do not know/not applicable
3. If yes, how is it performed?	Scavenger bird feeders/burial/by-products treatment unit/common garbage/do not know

## Results

### Initial examination

Regarding the percentage of animals hunted subject to meat inspection at a slaughterhouse or a game handling establishment, 59% (n = 56) of hunting areas send more than 50% of the carcasses to official inspection. From the remaining 41% (n = 39) of the hunting areas: 23% (n = 22) do not send any carcasses (all are for private consumption), and 18% (n = 17) ship <50%. The same percentage is observed in the question, “Are animals hunted in this hunting area subject to initial examination on-spot?” The 22 locations (23%) where none of the game is inspected also do not carry out the initial examination on-spot. About 60% (n = 57) carry out state the initial examination on-spot and 17% (n = 16) said that it is carried out sometimes.

In the case of the question “Are hunted animals eviscerated on the territory of the hunting area?” even if the initial examination is not carried out, 58% (n = 55) say that evisceration is in territories belonging to the hunting area, 23% (n = 22) affirm sometimes. In the remaining 19% of hunting areas (n = 18), evisceration occurs outside their territory.

The answers to the question “If it is performed, who does the initial examination?” were variable: In 39 hunting areas, the initial examination was performed by a trained veterinarian, in 32 by a veterinarian without specific formation, in 8 by a trained person/hunter and only 1 time by a hunter without the specific formation, and, in 22, the initial examination was not carried out.

### Hygiene requirements on-spot

Regarding the question about the location of the collection point (where the carcasses are eviscerated or/and the initial examination is conducted), 58% (n = 55) were inside the hunting area (some outside the hunting zone but within the territorial perimeter of the hunting area), 23% (n = 22) were outside the hunting area and in 19% not exist a specific spot/collection point. Of 78 collection points (82%) classified in relation to the hygiene requirements, 41 (53%) were paved with a permanent semi-hygienic soil (cement, tar, or tile), only 23 (30%) were covered, 50 (64%) had sufficient and potable water and 44 (56%) had electricity/light available.

### Biosecurity procedures on initial examination

Regarding the questions in category 3 of the survey, during the initial examination, the operator must respect the use of some protective material. Of the 95 hunting areas, in 24, the answer “not applicable” was marked (25%) to the questions “use of mask/disposable gloves/protective glasses/specific clothes or disposable suit,” either because the initial examination was not carried out or the game manager did not know the answer. About the remaining 71 answers, with regard to the use of disposable gloves, the majority (97%; n = 69) were using them. Thirty-four (48%) use masks, and only eight use protective glasses (11%). About the use of specific clothes or disposable suits, 56 answered yes (79%), five no (7%), and ten sometimes (14%).

### By-products disposal

Responding to the question, “Is the collection point cleaned and disinfected?” 29 collection points were not cleaned or disinfected (31%), 24 (25%) were just cleaned, but 21 (22%) were cleaned and disinfected. Concerning the questions about the disposal of by-products (“Carrying out proper disposal of by-products of hunted animals after evisceration and initial examination? If yes, how it is performed?”), 66 game managers said that this is done correctly (69%), being the majority destination the burial (64%; n = 47), followed by Scavenger bird feeders (22%; n = 16), common garbage (11%; n = 8), and only two use sometimes by-products treatment unit (3%).

## Discussion

In the past 10 years, some scientific publications related to post-harvest hygiene procedures for large game species have been published. Still, those that specify and analyze the collection points are rare. Most are related to the association of the harvest phases and conditions (e.g., environmental temperatures, the time between shotting and evisceration, time until carcasses colling and carcass transportation and storing) and other risk practices (e.g., bad anatomical shotting and consequent fecal contamination, or skinning process) that increase microbiological contamination of carcasses for consumption [5, 9, 13, 14]. This risk is greater in those carcasses where a meat inspection is not carried out. Therefore, private consumption of game meat not inspected can be considered one of the and most significant risks to the safety of consuming the large game meat [5, 10]. In our work, a median percentage of hunting areas was observed where all hunted animals are not inspected (23%) in slaughterhouses, nor is there an initial examination carried out in the field. Most of these areas are in the north and central-north of Portugal since they are located outside the risk zone delimited by Notice Nº1, where the initial examination is mandatory and carried out by a trained veterinarian. Thus, it is noted that although in some hunting areas, animals are eviscerated in their territory, there is no sanitary assessment to ensure the safety of the meat. The lack of hunters’ knowledge and the fact that non-eviscerated carcasses allow for transport mode of cleaner private transport. This is the reason why these two actions are sometimes avoided in the field, thus making self-consumption dangerous [10]. In recent publications in the Iberian Peninsula, where surveys of hunters were carried out, the tendency toward a large percentage of self-consumption is observed: 86% in a study about large game meat hunters in Portugal [15] and Spain [16]; in a similar survey, this value increases to 92.1% in consuming all game hunters, mostly large game consume.

In our study, in most of the hunting areas that were visited and that systematically carry out the initial examination on the spot, this procedure is performed by a veterinarian. This is essential data since these are technicians with greater training/specialization in the area and knowledge of public health prevention and promotion [17]. The number of areas where a trained person performs the initial examination is minimal, and without training is vestigial. It is an essential step toward promoting food safety, since, according to Mirceta *et al*. [18], the microbial count of carcasses increases when handled by untrained hunters.

One of the critical points in the game meat safety is to ensure a hygienic sanitary processing environment. Thus, it is essential to ensure some hygiene requirements at the collection points [19]. According to Vinhas [12], the most critical parameters in terms of structural requirements are potable water, light, and the floor but they need to be standardized [1, 19]. In our study, only the fact that there is no ceiling in most of the analyzed collection points; the vast majority have cleanable soil, potable water, and electricity. These requirements make it possible to optimize the hygiene of the infrastructures where the evisceration and the initial examination are carried out and to avoid cross-contamination [20]. Once the specialists affirm that the floor must be impermeable and cleanable. It is known that eviscerating and carrying out a sanitary assessment on the ground increases contamination of the carcasses, which can be contaminated with bacteria or soil [21]. Create a barrier between a clean and unclean environment or between carcasses and permit one effective cleaning service at the end of the sanitary evaluation and thus reduce the chance of cross-contamination in subsequent driven hunts. The location of the collection point in the hunting area is also critical. Since if this is within the hunting zone and there is no effective hygiene and correct by-products disposal, environmental contamination may remain after the hunting acts/driven hunts are contamination that can be a vehicle of pathogens for the animals that live in the hunting area. There is, thus, the acquisition of infectious pathogens by indirect contact between hunted and non-hunted animals.

To mitigate the risk of acquiring occupational diseases during the initial examination, biosecurity procedures and the use of protective material by operators are imperative. Systematically, disposable gloves (97%; n = 69) and suits or specific clothes (79%; n = 56) were the most used material. Large game species are vehicles and reservoirs of some zoonotic pathogens (viruses, bacteria, and parasites) [10], and the use of this material prevents the acquisition of these pathogens through direct contact, such as *Brucella* spp., *Erysipelothrix rhusiopathiae*, *Salmonella* spp., *Yersinia* spp., and *Coxiella burnetii* [22]. The fact that it is disposable also allows it to be disposed of correctly. It does not transport pathogens from the initial examination site to other locations, the operator himself being the vehicle of infectious diseases.

In this point, it is also essential to highlight the “human factor,” because the operators and food/meat handlers are responsible for personal hygiene and good meat handling [5, 13]. Not wearing gloves lead to an increase in microbiological counts on the surface of the carcasses [21].

The parameters analyzed in Category 4 of our survey about by-products disposal (“Carrying out proper disposal of by-products of hunted animals after evisceration and initial examination? If yes, how it is performed?”) are those that best fit the hygiene and biosafety requirements in all survey. Correct by-products disposal is essential to avoid the spread of certain diseases due to the access of domestic and wild animals to unfit meat and offal disposal sites [23]. To promote and increase hygiene criteria, the use of by-product treatment units should be encouraged, since, in our survey, this destination only occurs in two cases.

The parameter in this category that causes the most concern is that many collection points are not cleaned and disinfected after being used for evisceration and initial examination. Many of the spots do not have truly sanitized infrastructures, and as such, this good practice of hygiene needs to be improved. Failure to disinfect means that if dirt and infectious pathogens are maintained on site, there may be subsequent cross-contamination through indirect contact, regular use, and a place without hygienic conditions [12, 24].

In short, we know that the microbiological count in the large game carcasses’ surface increases due to unsuitable evisceration, structural and hygiene equipment not being clean, contamination of the carcass in the handling process, and the cross-contamination both by the environment and by the operators and contaminated carcasses themselves [21]. Thus, it is essential to characterize these conditions that allow an initial evisceration and examination to know the general panorama in Portugal and to understand the consequences of these in the game meat chain.

Self-consumption is a reality but must be carried out with awareness and control. Awareness of the need for an initial examination in the field must be a reality in areas of Portugal where this is optional. Training for hunters and people to perform the initial examination is necessary and imperative. A trained person has juridical responsibility, which is required to transmit and make people aware of. Limited awareness of food safety preventive measures and unhygienic handling might pose a foodborne infectious risk to hunters and their families [10, 19, 25, 24].

Nevertheless, with the results of our study, it is essential to recognize what must be improved to keep ensuring the safety of game meat and the protection of animal and human health [12, 25].

## Conclusion

There is an immediate need in all this problematic the standardization of the hygienic and biosecurity requirements of the collection points, and that requires uniform application of rules. The lack of an overview of all large game meat chains and the actual absence of microbiological criteria of this product lead to poor and maladjusted control. The present study proves that there still needs to be more rules for the entire process of handling products from game animals to reduce microbiological contamination and guarantee their food safety. This study was carried out with a small and convenient sample but suggested, as preliminary results, that it is more necessary for information and educational training about good hygiene practices and initial examination on-spot.

Training all those involved in the hunting area (hunters, game managers, authorities, etc.), creating rules that promote hunting food security and setting limits on the microbiological criteria of game meat are hot points to consider. This would be a crucial strategy and needed leverage to the economic development of hunting activities.

## Authors’ Contributions

ACA, BV, and MV: Conceptualization. ACA: Methodology, software, formal analysis, and writing–original draft preparation. MV and YV: Validation. ACA, ZR, and MPF: Investigation. ACA and MPF: Data curation. MV: Writing–review and editing and supervision. All authors have read, reviewed, and approved the final manuscript.
